# Bergmann Glia, Long-Term Depression, and Autism Spectrum Disorder

**DOI:** 10.1007/s12035-016-9719-3

**Published:** 2016-01-26

**Authors:** Adrian Andrzej Chrobak, Zbigniew Soltys

**Affiliations:** 1 Department of Neuroanatomy, Institute of Zoology, Jagiellonian University, Gronostajowa St. 9, Cracow, 30-387 Poland; 2 Faculty of Medicine, Jagiellonian University Medical College, Kopernika St. 21A, Cracow, 31-501 Poland

**Keywords:** Neuroglia, Plasticity, Radial glia, Excitotoxicity, Glutamate transporters

## Abstract

Bergmann glia (BG), a specific type of radial astrocytes in the cerebellum, play a variety of vital functions in the development of this structure. However, the possible role of BG in the development of abnormalities observed in individuals with autism spectrum disorder (ASD) seems to be underestimated. One of the most consistent findings observed in ASD patients is loss of Purkinje cells (PCs). Such a defect may be caused by dysregulation of glutamate homeostasis, which is maintained mainly by BG. Moreover, these glial cells are involved in long-term depression (LTD), a form of plasticity which can additionally subserve neuroprotective functions. The aim of presented review is to summarize the current knowledge about interactions which occur between PC and BG, with special emphasis on those which are relevant to the survival and proper functioning of cerebellar neurons.

## Introduction

The cerebellum is well known for its role in motor behavior. Moreover, there is growing evidence that this part of the brain is involved in various cognitive and affective processes [1, 2] and that its dysfunctions may be linked to various psychiatric disorders [3], including autism spectrum disorder (ASD) [4, 5].

The characteristic features of this syndrome such as deficit in social interactions or hypersensitivity to sensory stimuli [6] indicate the cerebral cortex as a potential locus of pathology. Nevertheless, significant anatomical pathology can also be seen in the cerebellum of ASD patients, including a reduction in the total volume, atrophy of the folia, [7, 8], and small foci of dysplasia [9]. Loss of Purkinje cells (PCs), the principal neurons in the cerebellum, is the most consistent cellular abnormality found in ASD [10, 11]. Importantly, this deficit has not resulted from decreased proliferation but from the loss of the PC in the later stages of development [12].

Bergmann glia (BG) is a type of radial glia specific for the cerebellum. While BG somata are localized in the Purkinje cell layer, their processes form a dense palisade extending through molecular layer of the cortex. Similar to protoplasmic astrocytes in other parts of the brain, BG processes cover synapses on Purkinje neuron dendrites [13], what suggests its participation in the regulation of synaptic transmission. Paradoxically, BG is the most ignored population of cerebellar cells. It is hard to find these cells in the textbook diagrams depicting the structure of the cerebellar cortex connections. Although each PC is linked to BG, the number of publications related to this glia is only 5–6 % of those on PC (according to Pubmed and Google Scholar search).

In this paper, we will present arguments in favor of the hypothesis that abnormalities in the interaction between BG and PC may lead to the emergence of autism spectrum disorders. First of all, we will briefly summarize our knowledge about the role of BG in the development and maintenance of the proper functioning of the cerebellum, with particular focus on glutamatergic transmission. Then, we will discuss the involvement of these cells in long-term depression (LTD). This phenomenon is usually regarded as a basic mechanism of cerebellar-dependent learning; however, it was also proposed that LTD serves a neuroprotective functions [14]. In the last part, we consider the possible causes of abnormal interactions between cerebellar cells and the effects of these anomalies for the functioning of the entire brain.

### Bergmann Glia and Purkinje Cell Interactions

BG play crucial role in regulation of cerebellum development—migration, cells’ maturation, and synaptogenesis [15–20].

On the other hand, BG requires continuous association with PC for the development of its own normal phenotype. For example, the presence of PC is necessary to maintain high levels of sn-glycerol-3-phosphate dehydrogenase—an enzyme involved in several metabolic pathways including lipid synthesis and energy metabolism [21]. Dynamic transformation of BG fibers and the expression of the glutamate transporter (GLAST) correlate with dendritic outgrowth and synapse formation of cerebellar PC [22]. Neuron-derived fibroblast growth factor 9, neuron-specific Delta/Notch-like EGF-related receptor, and sonic hedgehog protein are vital for mediating neuron-glia interaction and promote differentiation of BG and its GLAST expression [23–26].

Interactions between BG and neurons are very complex. Tables 1 and 2 present known elements of BG interactome. List of receptors reveals that these glial cells can react to numerous neurotransmitters, not only for those released by cerebellar neurons. On the other hand, action of BG gliotransmitters is not limited to PC. However, detailed discussion on these interactions is beyond the scope of this article.

**Table 1:** Bergmann glia receptors

Agonist	Bergmann glia receptors	References
Glutamate	Ca^2+^-permeable AMPAR, mGluR1, mGluR5	[27]
Purines	P2Y, P2X7	[28]
Serotonin	5-HT2A	[29]
Noradrenaline	α1A	[30]
Histamine	H1	[31]
Acetylcholine	M2	[32]
GABA	GABA_A_, GABA_B_	[33]
CRF	CRF-R1, CRF-R2α	[34]
Endothelin	ETB	[35]
T3	TRα1	[36]
BDNF	TrkB	[37]
Angiotensin II	AT1R	[38]
VEGFR	VEGFR-3	[39]
HGF	c-Met-IR	[40]
CGRP	CGRPR	[41]
Pathogen-associated molecules	Toll-like receptor 3	[42]
Melatonin	MT2	[43]
Chemokines	CCR1	[44]
PTN	PTPζ	[45]
Delta	Notch1, Notch2	[25, 46]
Delta-like 1		
DNER		
FGF	FGFR1	[47]
Shh	ShhR	[24]

**Table 2:** Substances released from Bergmann glia

Substances released from Bergmann glia	References
D-Serine	[48]
L-Glutamate	[49]
L-Glycine	[50]
GABA	[51]
Taurine	[52]
S100B	[53]
PTN	[45]
Gdf10	[24]
Il-1β	[54]

### Clearing of Glutamate

Causes of PC degeneration in ASD are still not fully understood. One of the most common causes of neuronal death is elevated extracellular glutamate level [55–57].

EAAT1 (GLAST) and EAAT2 (GLT-1) are astroglial transporters responsible for more than 80 % of total glutamate uptake in the CNS [58–60]. Reduction of GLAST and GLT-1 expression results in increased extracellular glutamate level and excitotoxicity leading to severe neurodegeneration [61].

GLAST stands for majority of glutamate transporters in the cerebellum; its amount is six times higher than GLT-1 and ten times higher than neural glutamate transporter—EAAT4 [62, 63]. The highest density of GLAST is presented in BG where it reaches ∼18,000 molecules per μm^3^ [62, 63]. GLAST-deficient mice present mild motor discoordination and increased cerebellar damage after cerebellar injury [64]. Lack of this transporter affects also PC innervation pattern, resulting in higher number of PC multiply innervated by climbing fibers (CF) in comparison to the wild-type mice [64]. Pharmacological blockade of these transporters prolongs PC α-amino-3-hydroxy-5-methyl-4-isoxazolepropionic acid receptor (AMPAR)-mediated excitatory postsynaptic current (EPSC) after both CF and parallel fiber (PF) stimulation and favors glutamate spillover into neighboring synapses [65]. Those observations indicate that GLAST plays an essential role in establishing morphological and functional one-to-one relationship between CF and PC [66].

Despite the fact that GLAST expression is sixfold higher than GLT-1 [63], GLAST knockout mice exhibit only minor motor discoordination [64] and reveal no significant alteration of CF-mediated EPSC in PC [67, 68]. Also, deletion of GLT-1 alone does not significantly affect cerebellar development [69]. However, knockout of both GLAST and GLT-1 disrupts cerebellar folium formation and results in prenatal death [70, 71]. This raises a possibility that GLT-1 may compensate for the downregulation or loss of the GLAST [71].

### GLAST in Direct Intracellular Signaling

GLAST may play an active role in direct intracellular signaling. In cultures of chick BG, glutamate triggers two temporally separated pathways affecting GLAST functions. In the “early” pathway, glutamate uptake by GLAST activation triggers Na^+^ influx that stimulates Na^+^/Ca^2+^ exchanger resulting in Ca^2+^ influx. This process leads to inhibition of GLAST translation (through mTOR pathway) and to downregulation GLAST expression in plasma membrane (through cytoskeletal re-arrangements). In the “late” pathway, glutamate binding to its receptors downregulates GLAST transcription in Ca^2+^/PKC-dependent manner [72].

What is intriguing in these findings is that glutamate action leads to downregulation of its own transporters, resulting in an inhibition of anti-excitotoxic system. The BG reaction to glutamate appears to be different to that observed in astrocytes, in which glutamate upregulates GLAST activity [73, 74]. However, these observations should be confirmed with further studies, at least in mixed cultures in which BG would be in contact with neural cells.

### AMPAR on BG

BG is involved in glutamate signaling not only by glutamate transporters but also by its expression of Ca^2+^-permeable AMPAR. This type of AMPA receptors contains GluA1 and GluA4 subunits. In the adult cerebellum, GluA1 subunit is exclusively localized to BG [75]. Conversion of those receptors into Ca^2+^-impermeable type by delivery of GluA2 gene results in retraction of the glial processes from PC. Retraction of the glial processes (with its glutamate transporters) results in increase in the distance between PC and BG membranes. This leads to similar consequences as GLAST (−/−) mutation in mice model: multiple innervation of PC by CF and impaired uptake of glutamate in both CF and PF synapses [76]. Consistently, overexpression of calcium-permeable AMPAR resulted in elongation of processes [77]. Further in vivo studies confirmed that mice knockout for AMPAR revealed similar retraction of BG processes and altered clearance of glutamate resulting in disrupted duration and decay of PF-evoked PC EPSCs. Furthermore, mice showed deficits in motor functions and motor learning [78].

Activation of AMPAR inhibits K^+^ conductance of the glia [79] likely due to the inhibition of gap junctional coupling [80]. In cultured chick cerebellar BG, AMPAR has also been shown to participate in downregulating transcription of GLAST [81].

**Fig. 1:**
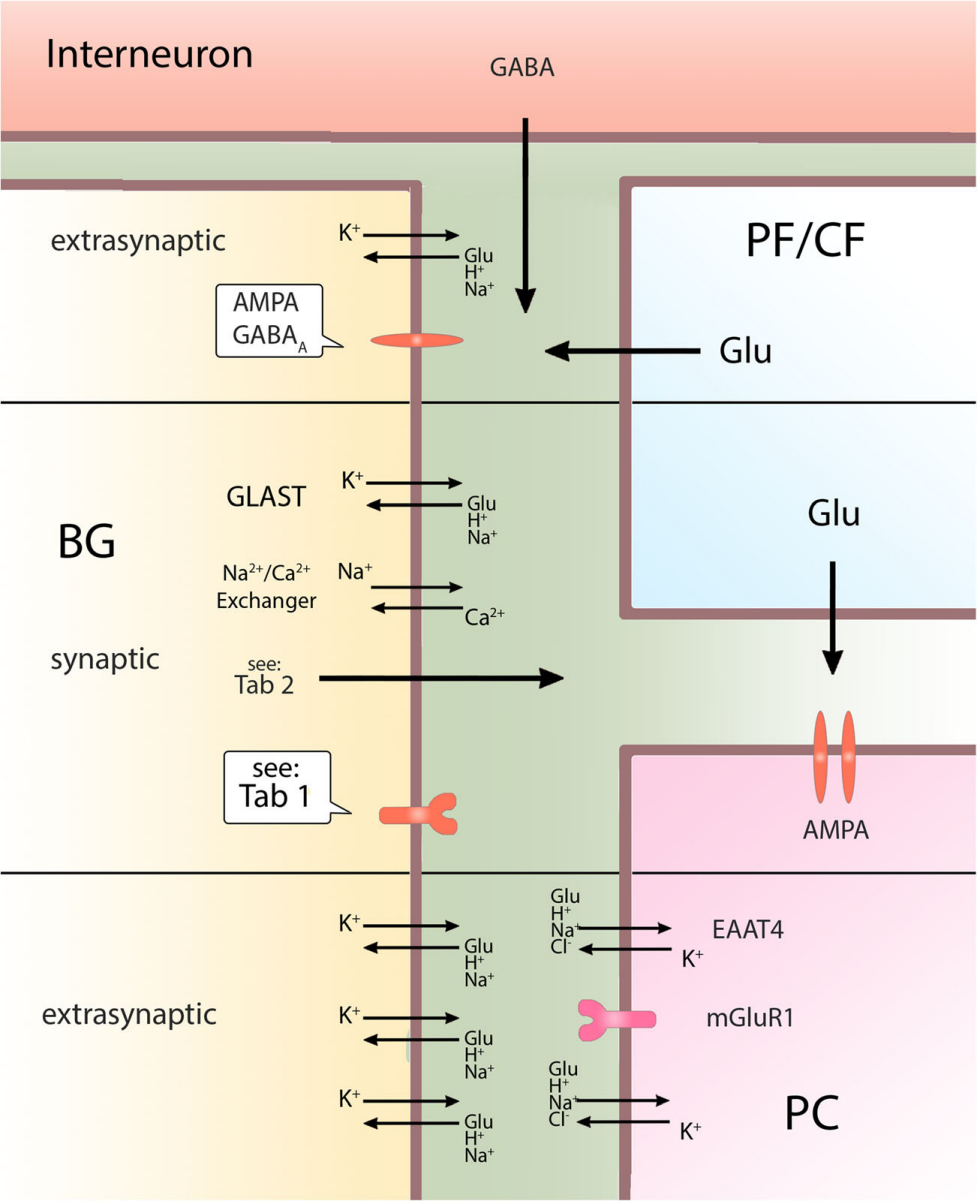
Bergmann glia interactions

Importantly, BG AMPAR are not activated “classically” by glutamate spillover from synaptic cleft but through specially dedicated extrasynaptic release sites (Fig. 1) [82–84], which exhibit a form of frequency-dependent plasticity due to the lack of fast vesicle recycling mechanism. Thus, during repetitive stimulation, exhaustible pool of vesicles in ectopic sites become depleted, resulting in the lack of glutamate released into BG-enriched AMPAR sites (Fig. 1) [85]. Repetitive stimulation of CF or PF at 0.1–1 Hz results in “long-term depression of neuron to glia transmission,” reducing Ca^2+^ currents in BG. This depression becomes persistent when PF stimulation reaches more than a few minutes, leading to inhibition of AMPAR Ca^2+^ currents [86]. This plasticity is not only activity-dependent but also input-specific because CF and PF inputs can be independently depressed [86].

### Cerebellar Long-Term Depression

LTD refers to the long-term attenuation of neurotransmission within the synapse after prolonged stimulation with an appropriate pattern. In the cerebellum, LTD occurs at excitatory synapses between PC and PF. Single PC receives excitatory glutamatergic input from hundreds of PF and from one CF. LTD is induced by simultaneous and repetitive stimulation from parallel and CF or by particular input from PF alone [87, 88]. LTD is expressed as long-term attenuation of glutamatergic transmission in PF-PC synapses by internalization of AMPAR in PC [87].

In 1997, Llinas et al. [14] proposed that LTD does not reflect motor learning process but a neuroprotective function defending PC from Ca^2+^ mediated excitotoxicity. One PC forms approximately 500 glutamatergic synapses with one CF [89] and up to 200,000 with PF [90], which situate PC at constant risk of Ca^2+^ influx from permanent synaptic bombardment. Overwhelming intracellular Ca^2+^ elevation leads to PC death or damage of its dendritic arbor. LTD occurs as a result of excessive excitation of PC and through the rise of intracellular Ca^2+^ leads to PKC activation, phosphorylation of AMPAR, and its internalization [14, 87]. Thus, LTD results in decreased responsiveness of PC to further glutamate stimulation [14]. However, this alternative view of LTD as a damage control mechanism did not achieve adequate attention. Despite experiments indicating that cerebellum-dependent learning such as eyeblink conditioning can actually be performed in mouse models lacking of LTD [91, 92], this phenomenon is still regarded mainly as a cellular substrate of cerebellar motor learning [93].

Shibuki et al. [94] demonstrated one of the first pieces of evidence that LTD requires BG, using mice devoid of glial fibrillary acidic protein (GFAP). This cytoskeletal protein is highly expressed in BG [94]. GFAP-deficient mice present impaired cerebellar LTD. This abnormality was not related to changes in synapse number or shape, altered protein expression, impairments in transmission of PF and CF, nor alterations in BG morphology [94]. The results indicated GFAP as a significant factor, needed for proper communication between BG and PC, enabling occurrence of LTD. GFAP may affect glutamatergic signaling because GFAP-null mice presented upregulation of both total and synaptosomal levels of EAAT2 protein in cerebellum [95]. It has been proposed that increased EAAT2 expression may limit LTD response by facilitating uptake of glutamate [95].

Importantly, stimulation of CF and PF at 1 Hz in order to evoke LTD in PC resulted in long-term depression of AMPAR currents in BG from both inputs [86]. Thus, during LTD, BG AMPAR is probably paradoxically cut off from their main source of glutamate. So, if the mechanism of AMPAR-derived downregulation of GLAST also exists in vivo, this would mean that during LTD, this process will be inhibited. This inhibition would avoid initiating GLAST reduction during states that threaten excitotoxicity and favor signaling involved in GLAST upregulation. Consistently, Balakrishnan et al. [85] suggests that glutamate is released from ectopic sites of fibers which are firing unusually slowly. Those fibers alert BG through AMPAR, resulting in encompassing of those faulty connections by BG processes. Probably, GLAST downregulation occurs in those synapses leading to strengthening of glutamate transmission.

Another mechanism involved in BG AMPAR regulation relies on the control of neuronal glutamate transporter, EAAT4, which represents one of the smallest fraction of glutamate transporters in cerebellum [63]. EAAT4 are expressed in PC, with the highest density in the dendritic and spine membranes facing BG (Fig. 1) [96]. EAAT4 is likely to affect extrasynaptic but not synaptic glutamate concentration [97]. Interestingly, higher expression of EAAT4 is inversely correlated with a degree of BG AMPAR activation after PF stimulation [97]. This could result in different BG AMPAR activation in regions with high or low expression of EAAT4, leading to differences in the level of encompassing PC synapses by BG processes in those regions [97]. This could affect regional differences in innervation of PC by CF, and GLAST regulation, which can correspond with the fact that variations in EAAT4 expression contribute to PC vulnerability to excitotoxic death [98]. The role of EAAT4 in regulating the structure of BG processes is enforced by the observation that in mice, its expression rises after birth, reaching maximum at the third week of age which corresponds to the time of maturation of BG ensheathment to PC synapses [97]. Additionally, EAAT4 are expressed closely to PCs’ mGluR1 receptors, which are crucial for LTD [99]. It has been shown that inhibition of EAAT4 facilitates cerebellar synaptic LTD, indicating its important role in plasticity [100].

Sasaki et al. [49] showed that the role of BG in LTD may be far more active than we thought. Photostimulation of BG in transgenic mice expressing channelrhodopsin-2 specifically in those cells resulted in an increase in extracellular K^+^, release of glutamate, and inducing LTD at PF-PC synapses. Glutamate released from BG activated mGluR1 on PC [49], which is essential for inducing this form of synaptic plasticity [101]. Processes of BG are located immediately adjacent to the mGluR1 on PC (Fig. 1) in the areas highly protected by neuronal glutamate transporters [102]. This arrangement raises a possibility that summation of glutamate from neuronal spillover and glial release may be required to overcome this protection to activate the mGluR1 [49] and enables glutamate originating from glia to act safer, by more direct action on the specific area covered with those receptors. A fact that stimulation of BG was sufficient factor to evoke LTD emphasizes important role of this cell in LTD and cerebellar functions.

PCs have specific orphan receptors GRID2 (δ2 glutamate receptor, GluD2). Mutations in *GRID2* gene were associated with ASD [103] (Table 3). These receptors are necessary for induction of LTD in the cerebellum (but not in the other parts of brain) [118]. Despite its name, GRID2 does not actually bind glutamate. Kakegawa and coworkers [119] have shown that D-serine may be an endogenous ligand for these receptors. It is derived mainly from BG after burst stimulation of PF in immature cerebellum [119]. Glutamate has been shown to activate BG serine racemase (which converts L-serine to D-serine) through AMPA receptors via glutamate receptor-interacting protein (GRIP) [120]. GRIP binds to serine racemase, enhancing its activity and release of D-serine [120]. Activation of GRID2 by D-serine results in rapid endocytosis of AMPA receptors and depressed excitatory postsynaptic currents in PC [119]. This process may stand for additional neuroprotective mechanism inducing LTD during early postnatal period.

**Table 3:** Genes associated with autism spectrum disorder expressed in Bergmann glia

Gene symbol	Gene name	Molecular function	Support for autism	Evidence of support	Number of gene association studies
*ANK2*	Ankyrin 2, neuronal	This gene encodes a member of the ankyrin family of proteins that link the integral membrane proteins to the underlying spectrin-actin cytoskeleton.	Rare single gene variant	Two de novo nonsense variants in the ANK2 gene have been identified in unrelated simplex ASD cases from the Simons Simplex Collection [104]	4
*APC*	Adenomatosis polyposis coli	The encoded protein is a tumor suppressor.	Genetic association	Rare mutation [105] and genetic association [106] of the APC gene has been identified with autism.	9
*BDNF*	Brain-derived neurotrophic factor	During development, promotes neuronal survival and differentiation of neurotrophic factor. Major regulator of synaptic transmission and plasticity at adult synapses in many regions of the CNS.	Functional	BDNF(+/−) mice displayed altered grooming, as well as hypolocomotion and increased turning behavior [107]. Abnormal BDNF serum levels in ASD cases have also been reported [108]	6
*CDH22*	Cadherin-like 22	Cell adhesion	Genetic association	Genetic association has been found between the CDH22 gene and autism in two large cohorts (AGRE and ACC) of European ancestry and replicated in two other cohorts (CAP and CART) [109]	4
*DAB1*	Disabled homolog 1 (Drosophila)	DAB1 serves as an intracellular adaptor for reelin signaling pathway	Genetic association	Significant reduction of Dab-1 mRNA was seen in superior frontal and cerebellar areas of autistic brains compared to control brains [110]	6
*GRID2*	Glutamate receptor, ionotropic, delta 2	Ionotropic glutamate receptors. GRID2 is strongly suggested to have a role in neuronal apoptotic death.	Rare single gene variant	Rare mutations in the GRID2 gene have been identified with ASD [103]. In particular, that study found two non-synonymous SNPs in GRID2 in 3 of 339 individuals with ASD.	6
*NRXN3*	Neurexin 3	Neurexins are a family of proteins that function in the vertebrate nervous system as cell adhesion molecules and receptors.	Rare single gene variant	Rare mutations in the NRXN3 gene, including deletions and missense variants, have been identified in patients with ASD [111].	9
*SLC1A1*	Solute carrier family 1 (neuronal/epithelial high affinity glutamate transporter, system Xag), member 1, EAAT3	Glutamate transporter.	Genetic association	Studies have found genetic association between polymorphisms of the SLC1A1 gene and autism [112].	11
*SLC35A3*	Solute carrier family 35 (UDP-N-acetylglucosamine (UDP-GlcNAc) transporter), member A3	UDP-N-acetylglucosamine transporter found in the golgi apparatus membrane.	Rare single gene variant	Deleterious compound heterozygous variants in the SLC35A3 gene were identified in eight patients from a large kindred presenting with autism spectrum disorder, arthrogryposis, intellectual disability, and epilepsy [113]	1
*UBE2H*	Ubiquitin-conjugating enzyme E2H (UBC8 homolog, yeast)	The encoded protein has ubiquitin-protein ligase activity.	Genetic association	Genetic association has been found between the UBE2H gene and autism in a French-Caucasian cohort [114].	1
*YWHAE*	Tyrosine 3-monooxygenase/tryptophan 5-monooxygenase activation protein, epsilon polypeptide	Regulation of a large spectrum of both general and specialized signaling pathways.	Rare single gene variant	A novel recurrent duplication involving the YWHAE gene was identified in two unrelated ASD cases [115]. Furthermore, the minimal region of overlap for cases with 17p13.3 microduplication, which includes autism as a phenotype, spans 72 kb and encompasses a single gene, YWHAE [116].	2

Based on sfari.org database and [117]

### Bergmann Glia and ASD

Reviewed studies indicate that BG is an important element of the system responsible in evoking LTD. Since BG functions and its participation in LTD exhibit neuroprotective effects, dysfunction of this system may contribute to development of various pathologies, including, *inter alia*, ASD.

Vulnerability of PC cells has been proposed to play a role in the etiology of ASD [57]. The hypothesis that dysregulation of glutamate transporters is one of the leading causes of neuronal dysfunction in those patients [56] corresponds with clinical observations which have shown that glutamate antagonists may reduce autistic symptoms [121]. ASD patients show that increased blood glutamate level correlated positively with increased glutamate level in the left cerebellum [122]. Several glutamate neurotransmitter system abnormalities have been found in cerebellum of individuals with ASD. Glutamic acid decarboxylase 65 and 67 kDa, the enzymes that catalyze decarboxylation of glutamate to GABA are significantly reduced in cerebellum and parietal cortex of ASD patients [123]. Postmortem samples revealed increased levels of EAAT1 and 2. Interestingly, EAAT1 protein increased threefold in autism cerebellum compared with controls. Those changes may reflect reaction due to elevated extracellular glutamate concentration. Furthermore, patients with ASD revealed significantly decreased level of AMPAR density, despite increased messenger RNA (mRNA) of GluA1. Those alterations may be associated with increased levels of proteins associated with AMPAR: GRIP and protein of band 4.1N [55].

4.1N and GluA1 reveal similar spatiotemporal pattern of expression. Both proteins are colocalized in intracellular organelles and cytoskeleton of BG [75], and they may form trimeric complex to regulate AMPAR localization and immobilization [75, 124]. Expression of 4.1N in BG becomes detectable when BG wraps PC [75]. A possible interpretation of this observation is that despite a global decrease in AMPAR density in ASD cerebellum, upregulated 4.1N and GluA1 may represent specific increase of AMPAR in BG.

Studies concerning participation of glia in cerebellar pathology in autism are scarce. Some of them indicate absence of glial hyperplasia [125, 126]; others reveal the presence of slight Bergmann gliosis and increase in GFAP level [127, 128]. Purcell et al. [55] identified increase in GFAP mRNA in patients’ cerebellum. Recent study reports over twofold higher expression of GFAP in cerebellum of individuals with ASD than in healthy controls providing evidence for astroglial reaction [129]. GFAP level has been found to be three times higher in cerebrospinal fluid (CSF) of ASD patients in comparison to healthy controls [130]. Additionally, increased incidence of GFAP autoantibodies was found in ASD [128]. Due the fact that GFAP seems to be associated with regulation of glutamate transporters, it is likely that dysregulation of GFAP expression may contribute to glutamate system abnormalities [95].

Also, a decrease in astrocytic aquaporin 4 (AQP4), a transmembrane water channel protein, has been reported in cerebellum of autistic patients [131]. It has been shown that this protein is crucial for recently described mechanism of brain parenchyma clearance (so-called glymphatic system [132]). According to experiments of Illif et al. [132], cerebrospinal fluid enters the parenchyma along perivascular spaces that surround penetrating arteries. Knockout in *Aqp4* gene results in ∼70 % reduction in efficiency of this mechanism [132]. Thus, it is possible that decreased aquaporin 4 in cerebella of ASD patients may contribute to disrupted interstitial solute clearance in this brain region, alternating extracellular levels of substances involved in neurotoxicity.

Transcriptional profiling of BG [117] revealed that those cells express several genes associated with ASD (Table 3). Many of them are directly or indirectly connected to glutamatergic transmission.

One of them is adenomatous polyposis coli (*APC*) gene, coding a multifunctional protein widely expressed in neurons and glial cells throughout the brain. APC protein is involved in plethora of processes, such as regulation of axon outgrowth, neuronal differentiation, and radial glial polarity [133]. Its heterozygous deletion or polymorphism is associated with ASD [106, 134]. APC protein regulates, among others, the canonical Wnt/beta-catenin signaling pathway [135], which seems to be involved in development and maturation of BG, which occurs simultaneously with PC dendritogenesis and synaptogenesis [136]. It is worth mentioning that mutations in *CTNNB1* (beta-catenin) or *WNT* genes also are linked to this disease [137, 138].

Insight on the role of APC in the cerebellum was brought by Wang et al. [133] who proved the crucial and selective role of this protein in maintaining the morphology and function of BG. Mice in which *APC* gene was inactivated in GFAP-expressing cells showed marked abnormalities in BG morphology (but not in astrocytes in other parts of the brain). Since then, radial fibers of BG shortened significantly with a marked reduction of branching collaterals. BG bodies translocated into the molecular layer, loss the contact with the pia, and transformed into stellate-shaped cells. During middle age, PC loss was significant, especially in lobules VI, VII, and VIII where disruption of BG morphology was most severe (which actually overlap with the most disrupted lobules in neuropathology of autism [10]). Wang et al. [133] suppose that degeneration of PC is caused by deprivation of glial control of glutamate clearance.

Anomalies in BG found in mouse models are similar to those reported in study of Wegiel et al. [9]. In some of the autistic brains, they observed dispersion of BG somata within the molecular layer and total loss of its vertical fibers. Morphologically, these cells were similar to cortical astrocytes. Simultaneously, underdevelopment of PC was observed in dysplastic regions. Unfortunately, the genetic background of analyzed cases was not determined in this study. As far as we know, there is no postmortem studies evaluating BG in humans with disrupted APC.

## Conclusions

Autism spectrum disorder can be caused by a variety of genetic and environmental factors, which concomitant action results in structural and functional abnormalities in various parts of the brain. In the cerebellum, the most marked pathological changes seen are underdevelopment or degeneration of PC. We have presented above data pointing to the hypothesis that abnormalities in PC can be caused by lack of appropriate support from BG. Like astrocytes, BG can protect PC from excitotoxicity by the clearance of glutamate. Moreover, cerebellar LTD (seen as a neuroprotective mechanism) requires BG to occur. Recent findings which revealed that disruption of BG functions leads to PC degeneration indicate a previously overlooked causative mechanism for ASD phenotypes. We suppose that therapies targeting BG can be efficient for treating some pathological features of ASD.
